# Case Report: Infant With Congenital Adrenal Hyperplasia and 47,XXY

**DOI:** 10.3389/fgene.2021.808006

**Published:** 2022-01-12

**Authors:** Sophia Q. Song, Andrea Gropman, Robert W. Benjamin, Francie Mitchell, Michaela R. Brooks, Mary P. Hamzik, Kira Sampson, Ritika Kommareddi, Teresa Sadeghin, Carole A. Samango-Sprouse

**Affiliations:** ^1^ Department of Research, The Focus Foundation, Davidsonville, MD, United States; ^2^ Division of Neurogenetics and Developmental Pediatrics, Children’s National Health System, Washington, D.C., DC, United States; ^3^ Department of Neurology, George Washington University, Washington, D.C., DC, United States; ^4^ Pediatric Endocrinology, Duke Children’s Hospital, Durham, NC, United States; ^5^ Department of Pediatrics, George Washington University, Washington, D.C., DC, United States; ^6^ Department of Human and Molecular Genetics, Florida International University, Miami, FL, United States

**Keywords:** congenital adrenal hyperplasia, klinefelter syndrome, case report, XXY (aneuploidy of klinefelter syndrome), neurodevelopment

## Abstract

Congenital adrenal hyperplasia is a group of autosomal recessive disorders in which enzymes in the cortisol biosynthesis pathways are disrupted by gene mutations. The most common form of congenital adrenal hyperplasia, caused by 21-hydroxylase deficiency, is characterized by decreased cortisol and aldosterone synthesis and excessive androgen production. Adult height is often compromised in affected patients. Intellectual capability remains intact in patients with congenital adrenal hyperplasia caused by 21-hydroxylase deficiency, based on previous studies. 47,XXY (KS) is a sex chromosomal aneuploidy that manifests with hypergonadotropic hypogonadism, tall stature, and variable intellectual and behavioral dysfunction. This clinical report describes an infant with 21-hydroxylase deficiency congenital adrenal hyperplasia and 47,XXY. The results of his neurodevelopmental, endocrine, neurological, and physical therapy evaluations during his first 22 months are included and were normal. This is the first published case investigating the neurodevelopmental profile of a patient with the combination of these two genetic disorders.

## Introduction

Congenital adrenal hyperplasia (CAH) describes a family of autosomal recessive diseases caused by mutations in genes encoding enzymes in the cortisol biosynthesis pathway. The clinical and biochemical manifestations of CAH are quite variable. The most common form of CAH, making up more than 95% of congenital adrenal hyperplasia cases, result from 21-hydroxylase deficiency (21OHD), due to loss of function mutation in *CYP21A2* (Speiser et al., 2010). 21OHD is characterized by lowered cortisol and aldosterone synthesis and androgen excess, the latter from marked adrenocorticotropic hormone (ACTH)-stimulated androgen production. Classic CAH from 21OHD occurs in 1:10,000 to 1:20,000 live births depending on ethnicity and is further separated into salt wasting and simple virilizing forms (Therrell, 2001). Completely inactive *CYP21A2* results in the classical salt-wasting phenotype, whereas mutations that retain 21OH activity and aldosterone production produce the simple virilizing form. Intellectual disability is not a typical feature of classical 21OHD.

Klinefelter syndrome (KS) or 47,XXY is the most common sex chromosome aneuploidy, occurring in 1:450 to 1:660 live males births (Bojesen et al., 2003; Morris et al., 2007). The diagnosis of KS is made by karyotype and requires at least one extra X chromosome. The most common karyotype in KS is 47,XXY. The additive X chromosome affects multiple organ systems. KS is characterized by tall stature and hypergonadotropic hypogonadism, which contributes to osteoporosis and low fertility. While males with KS typically demonstrate intellectual capability within normal limits, this population has an increased risk of language-based learning disabilities (LLD) and attention deficit hyperactivity disorder (ADHD). Many patients with 47,XXY remain undiagnosed in their lifetimes (Bojesen et al., 2003), however, more individuals are being diagnosed from noninvasive prenatal screening (NIPS) that detects fetal aneuploidy (Howard-Bath et al., 2018). In the United States, NIPS is offered to all expectant mothers, and is typically performed between 11 and 14 weeks of gestation. Fetal cell-free DNA (cfDNA) is obtained from the mother to assess risk for an aneuploid fetus; positive or borderline results require confirmation via diagnostic tests, such as amniocentesis.

We describe a patient with 21OH deficiency CAH and 47,XXY. The estimated incidence of CAH in conjunction with KS is approximately 1:7,500,000. Patients with these rare presentations have been described several times in the literature. The first case described a male who was diagnosed at birth with CAH and diagnosed with 47, XXY when 10 years old (Yamaguchi et al., 1994). Parker et al. (2006) reported a boy with positive newborn screening of CAH and later diagnosis of 48,XXXY/47, XXY mosaicism after concerning physical exam that included poor weight gain, microcephaly, and mild developmental delay. Another study reported a boy who was diagnosed with CAH at 2 years old but evaded evaluation for 47,XXY until he was 18 years old, primarily due to low testicular volume (Zanella et al., 2018). We present a case of a young male with the salt-wasting phenotype of caused by 21OHD CAH and 47,XXY. To our knowledge, this is the first report describing prenatal diagnosis of CAH with KS, and explores the patient’s neurodevelopmental profile from birth to 22 months of age. . We also consider the intriguing interaction between the expected androgen excess seen in CAH with the hypogonadism seen in KS.

## Case Description

### History

Our patient’s conception resulted from an intracytoplasmic sperm injection along with *in vitro* fertilization (ICSI-IVF) pregnancy with a frozen embryo transfer that was preserved from prior harvesting in 2014 after discovery of oligospermia. Pre-implantation genetic diagnosis was not considered. He is the product of a 40-weeks gestation to a healthy 34-year-old gravida-3, para-4 Middle Eastern female and her 36-year-old consanguineous mate. Mother and father are first cousins. 47,XXY was identified using noninvasive prenatal screening (NIPS) and confirmed with amniocentesis at 13 weeks gestation, which also diagnosed him with 30 kb homozygous deletion of *CYP21A2* CAH. He was delivered via a planned Cesarean section, and birth weight was 3.97 kg (8.75 pounds). Physical examination was remarkable for right sided cryptorchidism. He was discharged to home at 2 days of life without incident.

At 3 days of life, the patient was evaluated by pediatric endocrinology. Laboratory workup revealed markedly elevated serum ACTH and 17-hydroxyprogesterone levels and genetic confirmation of *CYP21A2* mutation. He was started on hydrocortisone (13 mg/m^2^/day), Florinef (0.05 mg q12), and NaCl (4.8 mEq/kg/day). He has had routine appointments with pediatric endocrinology since birth.

At 6 weeks of life, the patient was admitted for parainfluenza and was treated with stress doses of steroids. He did not have electrolyte abnormality or evidence of adrenal crisis with this admission. At 13 months of age, he had surgical repair of a right-sided inguinal hernia and right orchiopexy. He received stress coverage with hydrocortisone and was clinical stable throughout his short stay in the hospital.

A three-generation pedigree was completed during the initial evaluation. Mother’s first pregnancy with the same father resulted in fraternal twins delivered by Cesarean section at term, one male and one female whowere the products of IVF. The male was diagnosed at birth with CAH from 21OHD, at but does not have KS. He has developed normally, remains on physiologic hydrocortisone and fludrocortisone, and has also had routine visits with pediatric endocrinology. The female twin is healthy and carries no diagnosis. The secondpregnancy was a singleton girl born by vaginal delivery. She is healthy and carries no diagnosis. There is no other significant family history reported.

### Neurodevelopment

**FIGURE 1:**
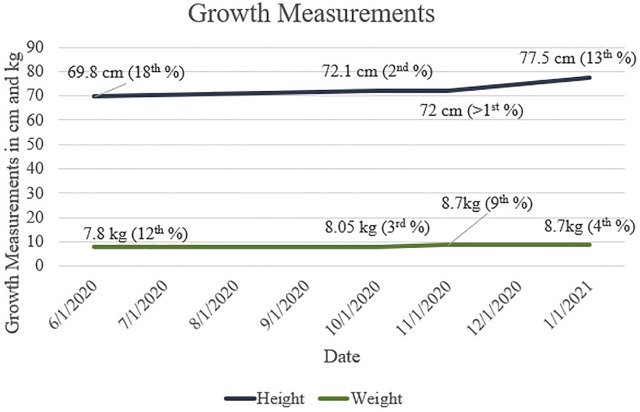
Height and Weight measurements throughout first and second years of life for patient with CAH and 47,XXY. Growth percentages are based on WHO (Boys, 0/2 Years) data.

Growth measurements for our patient are presented in Figure 1. Motor progression is age appropriate, and he was discharged from physical therapy. The patient rolled at 3 months, sat independently at 6 months, crawled at 8 months, and walked at 12 months. His first words spoken were at 6 months of age, and he had 2-word combinations at 22 months. He is currently receiving speech and language services twice a week. The patient understands both Arabic and English.

Comprehensive neurodevelopment at two time points during infancy was evaluated with the following measures: Preschool Language Scale, Fifth Edition (PLS-5), Early Language Milestone Scale, Second Edition (ELM-2), and Bayley Scales of Infant and Toddler Development, Fourth Edition. These results are presented in Table 1.

**TABLE 1:** Neurodevelopmental scores during evaluation at 9 and 16 months.

Age	PLS		ELM		Bayley		
	Auditory comprehension	Expressive communication	Auditory expressive	Auditory receptive	Cognitive (MDI)	Motor (PDI)	Language
9 months	114 (82nd %)	109 (73rd %)	100 (50th %)	104 (60th %)	85 (16th %)	84 (14th %)	89 (23rd %)
16 months	100 (50th %)	105 (63rd %)	98 (45th %)	75 (5th %)	105 (63rd %)	106 (66th %)	79 (8th %)

Physical therapy evaluation of our patient at 18 months was essentially normal, with a few abnormal findings. His examiner noted mild right torticollis and mild shoulder elevation and protraction, mild hypotonia and fifth finger clinodactyly. He had slightly low-set ears and a smaller right leg. Neurological examination revealed intact cranial nerves and bilateral sensation in upper and lower extremities.

## Discussion

This case explores the unique interaction between two distinct genetic disorders, congenital adrenal hyperplasia and KS from 47,XXY. We hypothesize that these two disorders could counterbalance each other, and potentially result in a normal phenotype. This report informs medical providers of potential treatment and clinical management for similar cases in the future.

Our patient was diagnosed antenatally with CAH from deficiency of 21-hydroxylase, which is typically characterized by adrenal insufficiency and androgen excess. His adrenal insufficiency was confirmed (Table 2), and he was started immediately on glucocorticoid and mineralocorticoid replacement. Although at risk of hyperandrogenism, he had normal penis size, no signs of adrenarche, and an undescended righttesticle. It should be noted that hyperandrogenism can be quite difficult to appreciate in male infants and often manifests later in life with precocious hair development, accelerated linear growth, and short stature. Cryptorchidism is not typically described in CAH (Howard-Bath et al., 2018).

**TABLE 2:** Laboratory results.

Labs	Value			References range for Prepubertal children
	September 2019	May 2020	November 2020	
Androstenedione	—	<10 ng/dl	<10 ng/dl	<10–17
Total Testosterone	—	2.7 ng/dl	<2.5 ng/dl	<2.5–10
17-Alpha-Hydroxyprogesterone	9,396 ng/dl	819 ng/dl	97 ng/dl	<91
Plasma Renin Activity	—	—	18 ng/mL/h	1.4–7.8
Adrenocorticotropic hormone (ACTH)	1,029 pg/ml	—	10 pg/ml	9–52 pg/ml^a^

a Reference range for blood sample taken early in the morning from 7:00–10:00 AM.

Our patient was also diagnosed with 47,XXY before birth, which is the most common karyotype in KS. KS is associated with tall stature and hypogonadism (Simpson et al., 2003). Tall stature in this syndrome is likely multifactorial, and in part results from overexpression of the *SHOX* stature gene on the extra X chromosome. Hypogonadism may manifest as cryptorchidism and/or micropenis. It is possible that higher adrenal androgen levels from CAH had a positive impact on penile growth and prevented bilateral cryptorchidism.

Neurodevelopmental findings in individuals with 47,XXY have been well-documented, though data are lacking in patients with coexisting CAH. Males with 47,XXY show wide variability in their cognitive abilities, though most fall within the normal IQ range (Yamaguchi et al., 1994). Children and adolescents with appropriately treated CAH exhibit normal cognitive and executive function (Parker et al., 2006; Messina et al., 2020a). However, parental reports indicate increased social problems on the Childhood Behavior Checklist (Zanella et al., 2018; Messina et al., 2020b). This may happen in 47,XXY as well, so we wondered how the potential mitigation of a supernumerary X with CAH could affect this aspect. To the best of our knowledge, none have addressed the neurodevelopmental phenotype of the two synchronously occurring disorders.

The patient exhibited normal gross and fine motor skills with slightly decreased muscle tone, the latter finding likely due to his diagnosis of KS. Untreated males with 47, XXY typically exhibit motor delays and global motor deficits. Early treatment with androgen has been shown to improve body composition in infants with 47,XXY, and may enhance truncal and gross motor control (Simpson et al., 2003; Davis et al., 2019). In our patient, the higher androgen levels caused by CAH may have acted similarly to hormonal treatment for his KS, and may explain why he performed within the normal range of motor competency.

His speech and language capabilities are within normal range and are currently supplemented with weekly speech therapy. This was started due to initial concern for speech delay. The higher androgen levels caused by CAH could have protected against the speech and language delays that may be exhibited in patients with 47,XXY. He said his first words early, at 6 months of age and reached the 2-word combination milestone at the neurotypical age of 22 months. The discrepancy between these language assessments may be due to fatigue or non-compliance during testing. The patient’s intellectual abilities are also in the normal range. Our findings are consistent with the literature available in both genetic disorders, as this patient’s cognition is comparable to neurotypical infants of the same age. It is recommended that this patient continues yearly follow-up neurodevelopmental evaluations as well as routine endocrine evaluations. Services should be continued as needed until there is little discrepancy between his skills and his neurotypical peers.

The major limitation of our study was the lack of progressive laboratory results and inconsistent timing of growth measurements. The patient has also not yet undergone neuroimaging, such as functional magnetic resonance imaging (fMRI), or electroencephalography (EEG) scans to determine neuronal differences, though this data may become available as he becomes older.

The age appropriate phenotype of this male suggests benefit of the androgen excess from CAH, which offsets the deficiency in 47,XXY. Continuation of neurodevelopmental observation of this patient is necessary to expand on this unique case.

## Data Availability

The raw data supporting the conclusion of this article will be made available by the authors, without undue reservation.
